# β‐Klotho sustains postnatal GnRH biology and spins the thread of puberty

**DOI:** 10.15252/emmm.201708180

**Published:** 2017-08-04

**Authors:** Micheline Misrahi

**Affiliations:** ^1^ Medical Faculty Hospital Bicêtre Université Paris Sud Le Kremlin Bicêtre France

**Keywords:** Genetics, Gene Therapy & Genetic Disease, Metabolism

## Abstract

Hypogonadotropic hypogonadism is a syndrome found to be isolated (IHH) or associated with anosmia, corresponding to the Kallmann syndrome (KS). It comprises a defect in gonadotropin‐releasing hormone (GnRH) secretion and absent or delayed puberty. Genetic causes have been identified with a high genetic heterogeneity. Fibroblast growth factor receptor 1 (FGFR1), a tyrosine kinase receptor, was one of the first genes whose mutations were identified as causative in KS. FGFR1 is responsible for the formation of the GnRH neuron system. Studying patients has not only allowed the identification of new etiologies for this syndrome but also helped to unravel the signaling pathways involved in the development of GnRH neurons and in GnRH control and function. The FGF21/FGFR1/Klotho B (KLB) signaling pathway mediates the response to starvation and other metabolic stresses. Preventing reproduction during nutritional deprivation is an adaptive process that is essential for the survival of species. In this work, Xu *et al* (2017), using a candidate gene approach, provide a description of the essential role played by this pathway in GnRH biology and in the pathogenesis of IHH and KS. They establish a novel link between metabolism and reproduction in humans.

The hypothalamic secretion of GnRH by GnRH neurons is essential for the onset and maintenance of reproduction. GnRH induces synthesis and secretion of follicle‐stimulating (FSH) and luteinizing (LH) hormones by the pituitary, themselves acting on the gonads to induce sex steroid production. A defect in GnRH secretion or action results in congenital hypogonadotropic hypogonadism (CHH), a rare genetic disorder characterized by lack of puberty and infertility. CHH exists in two forms: idiopathic hypogonadotropic hypogonadism (IHH) with anosmia (KS) or with a normal sense of smell (normosmic IHH: nIHH), which are both associated with deficient GnRH secretion. In addition, metabolic defects are present, however considered as secondary to sex steroid deficiency as usually improved by steroid treatment. IHH and KS are genetically heterogenous with more than 30 causative genes identified to date (Boehm *et al*, 2015).

*FGFR1* has been one of the first two genes described as responsible for KS. This gene is also found mutated in IHH showing the proximity of the two syndromes. FGFR1 is a member of the FGFR family of transmembrane receptors with intrinsic tyrosine kinase activity. FGFR1 is important for multiple biological processes, including cell growth and migration, organ formation, and bone growth. *FGFR1* is highly expressed in central nervous system tissues and is involved in the development and migration of GnRH neurons (Boehm *et al*, 2015).

FGFs are growth factors that bind to FGFRs and act in a paracrine or endocrine manner. They control multiple biological processes such as proliferation, survival, migration, and differentiation of a variety of cell types. FGFs are involved in the formation and maintenance of GnRH neurons (Owen *et al*, 2015). FGF ligands may have paracrine or endocrine activity. Compared to paracrine FGFs, endocrine FGFs have poor affinity for their cognate FGFRs. They overcome this deficiency through the parallel binding to α/β Klotho coreceptors expressed in their target cells (Goetz *et al*, 2012; Owen *et al*, 2015). Klotho (*KL*) is a transmembrane protein discovered in 1997. The name of the gene comes from Greek mythology. Klotho means “spinner” in Greek. Klotho was one of the Three Fates of Destiny and responsible for “spinning the thread of life”. Mutation of the mouse *klotho* gene leads to a syndrome resembling aging and shortens lifespan. β‐Klotho *(KLB)* has been identified by homology with the KL gene (Ogawa *et al*, 2007). It is abundantly expressed in metabolic tissues, especially adipose tissue. FGF21 is an endocrine FGF mainly secreted by the liver that regulates major metabolic processes such as glucose and lipid metabolism and decreases body weight. Endogenous FGF21 plays a role in mediating the physiological response to starvation and a variety of other metabolic stresses (Owen *et al*, 2015). FGF21 signals primarily on a tissue‐specific manner through the β‐Klotho/FGFR1c receptor complex (Ogawa *et al*, 2007).

Because altered metabolism is associated with altered reproduction, Xu *et al* (2017) suspected that FGF21/KLB/FGFR1 signaling was involved in the pathogenesis of CHH. By using a candidate gene approach to study CHH and many different molecular and cellular approaches *in vitro* and *in vivo*, they convincingly demonstrate that β‐Klotho is involved in postnatal GnRH biology (Xu *et al*, 2017). Compared to its homolog KL, KLB “spins the thread of puberty”.

This genetic study of patients has made a leap in reproductive research allowing, in particular, to unravel multiple steps of FGFR1 signaling and function *in vivo,* altered in CHH.

The authors previously identified FGF8b as a key ligand for FGFR1c during embryonic development (Pitteloud *et al*, 2007). Specifically, they studied a patient with KS displaying an *FGFR1* loss‐of‐function mutation, L342S, which alters FGF8b binding. The patient also had metabolic phenotypes with severe insulin resistance.

Pitteloud *et al* then identified missense mutations in *FGF8* in IHH probands with variable olfactory phenotypes (Falardeau *et al*, 2008). Furthermore, mice homozygous for a hypomorphic *Fgf8* allele lacked GnRH neurons in the hypothalamus and exhibited nasal cavity developmental defects and olfactory bulb dysgenesis, a phenotype similar to that observed in the *Fgfr1* conditional knockout mouse (Chung *et al*, 2008). Heterozygous mice showed substantial decreases in the number of GnRH neurons and hypothalamic GnRH peptide concentration. The authors conclude that *FGF8* is implicated in GnRH deficiency in both humans and mice and report the exquisite sensitivity of GnRH neuron development to reductions in FGF8 signaling during development. Mutations in FGFR1 and FGF8 account for ~12% of cases of CHH (Falardeau *et al*, 2008).

Thus far, the natural ligand of FGFR1 in postnatal biology was unknown. The expression of *FGF8* is restricted to embryonic development. FGF21 has been identified as a major peripheral and central metabolic regulator (Owen *et al*, 2013). Because of the link between metabolism and reproduction, Xu *et al* (2017) hypothesized that a defect in the FGF21/FGFR1/KLB pathway may underlie GnRH deficiency in humans and rodents.

In this work, Pitteloud *et al* show that the CHH‐associated FGFR1 mutation p.L342S leads to a decreased signaling of the metabolic regulator FGF21 by impairing the association of FGFR1 with KLB, the obligate coreceptor for FGF21. Interestingly, the KS patient also had metabolic defect.

The change of FGFR1 signaling between embryonic and adult life is operated by the loss of expression of the paracrine factor FGF8 and the expression of an endocrine ligand, FGF21, together with a mandatory expression of the FGFR1 coreceptor KLB in a tissue‐specific way. *Klb* is expressed in the postnatal hypothalamus. KLB enhances FGF21‐FGFR1c (an isoform of FGFR1) binding and hence promotes FGF21 signaling by simultaneously tethering FGF21 and FGFR1c to itself through two distinct sites. Furthermore, the competitive binding of FGF8 and β‐Klotho to the same site of FGFR1 will favor binding to endocrine FGF21 and inhibit paracrine FGF8 binding and signaling (Goetz *et al*, 2012). Indeed, the binding of KLB involves a conserved hydrophobic groove in the immunoglobulin‐like domain III (D3) of FGFR1c (Goetz *et al*, 2012). Interestingly, this hydrophobic groove is also used by paracrine FGF8 ligands for receptor binding. Amino acid L342, highly conserved across species, is a key constituent in the hydrophobic groove of D3 (Fig 1; Pitteloud *et al*, 2007; Goetz *et al*, 2012). This leucine accounts for the unique binding specificity determinant of FGF8b for the c splice isoforms of FGFR1–3.

**Figure 1:**
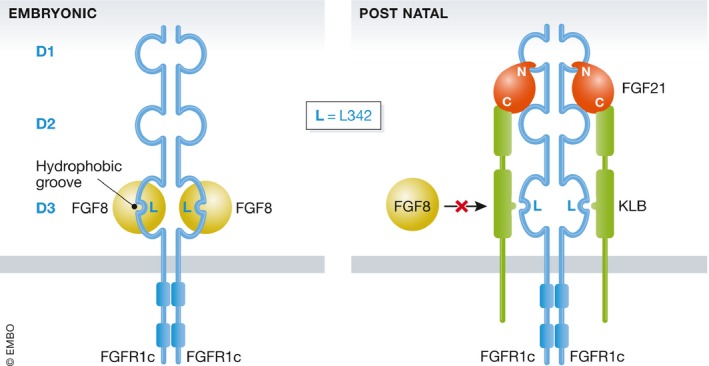
Binding of paracrine FGF8 and endocrine FGF21 to FGFR1c The binding of paracrine FGF8 to FGFR1c involves the conserved hydrophobic groove in the immunoglobulin III domain (D3) of FGFR1c including L342 (L). The binding of endocrine FGFs necessitates an absolute coreceptor, KLB for which binding to FGFR1c also involves the hydrophobic groove of D3 and L342. There is thus a competitive binding with FGF8.

To know whether the FGF21/KLB/FGFR1 signaling pathway was involved in GnRH deficiency in humans, a candidate gene approach in 334 patients with CHH was performed. The majority of patients also exhibited metabolic syndrome. While no mutation of FGF21 was identified, mutations of *KLB* were detected. Seven heterozygous variants were identified in 13 CHH probands including six missense variants and one in frame deletion in seven unrelated patients. The variants have decreased signaling, ligand affinity binding, or decreased expression *in vitro*. Wide phenotypic variability was observed from severe to mild forms such as CHH with reversal or fertile eunuch syndrome. Variable expressivity and incomplete penetrance were found in the families. This suggests that other genes or environmental factors may contribute to the phenotype. Indeed, 35% of patients were found to carry a supplementary heterozygous mutation of *FGF8, PROKR2*, and *FGFR1*, predicted or shown to be deleterious. A more severe phenotype was observed in the families with two genes mutated, and partial GnRH deficiency was observed in patients with *KLB* mutations alone. This is compatible with an oligogenic model of inheritance, which might explain the difference in phenotype and expressivity of the syndrome in a single family. The same group has previously shown that IHH can be caused by the combination of genetic defects. In total, 4% of patients had heterozygous *KLB* mutations. The majority of CHH patients with *KLB* mutations exhibit metabolic defects; 17% of CHH patients carry mutation(s) in FGF21/KLB/FGFR1 pathway, either as monoallelic or digenic combinations. Mutational analysis by next‐generation sequencing will allow uncovering a proportion of patients with oligogenicity and may lead to greater accuracy in phenotypic predictions. Oligogenicity also has implications for genetic counseling regarding IHH patients and their family.

*In vivo* models were studied by Xu *et al* (2017) to confirm the pathogenicity of the mutations detected. Complementation studies in *Caenorhabditis elegans* where the two homologs *klo‐1* and *klo‐2* were depleted showed that the mutants failed or had a decrease ability to rescue the cyst phenotype of the double‐deleted mutant. In addition, Xu *et al* (2017) studied the reproductive phenotype of KlbKO mice and show that they exhibit disrupted estrous cycles, blunted LH levels at estrus stage, and impaired fertility due to a hypothalamic defect. *Klb* ^−/−^ mice do not have abnormalities in GnRH neuron differentiation. There is a normal GnRH vesicular pool at the nerve terminals. *Klb* ^−/−^ mice respond to GnRH and kiss stimulations. This excludes a pituitary defect and suggests that GnRH neurons are present and can respond to stimulation. These results support an implication of KLB in postnatal hypothalamic GnRH secretion, consistent with a contribution of KLB to the central regulation of reproduction (Fig 2). Interestingly, the heterozygous KlbHET mice had a similar phenotype. A mechanism of haploinsufficiency is thus conceivable in the patients with heterozygous loss‐of‐function mutations of KLB.

**Figure 2:**
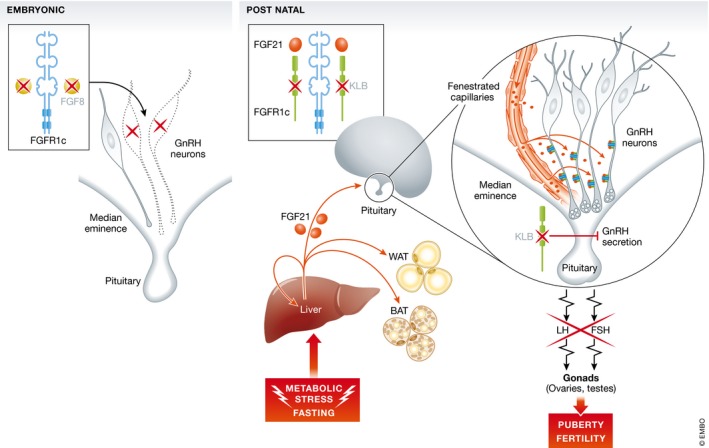
Consequences of paracrine or endocrine FGF deficiencies during embryonic and postnatal life on the neuroendocrine control of reproduction During the embryonic development, the paracrine FGF8 binds FGFR1c. Deficient FGF8 signaling results in absence of GnRH neuron development in the hypothalamus. During postnatal life, the endocrine FGF21, secreted under the influence of metabolic stimuli, acts on the liver and adipose tissue (WAT, white adipose tissue; BAT, brown adipose tissue) and reaches the hypothalamus through fenestrated capillaries (FC) of the median eminence (ME) or of the organum vasculosum of the lamina terminalis. FGF21 binds FGFR1c and the obligate coreceptor KLB. Deficient KLB is associated with normal embryonic GnRH neuron development but with a block in GnRH secretion during postnatal development. There is an altered secretion of LH and FSH by the pituitary (P) leading to altered puberty and fertility.

At the molecular level, Xu *et al* (2017) show that FGF21 stimulates neurite outgrowth in mature immortalized GnRH neurons *in vitro* and induces GnRH secretion/release in median eminence (ME) explants *ex vivo*. These results raise the possibility that peripheral FGF21 modulates GnRH secretion by acting directly on GnRH neuroendocrine terminals in the ME and suggest a novel role for FGF21 in controlling fertility by modulating GnRH neuron structural plasticity.

By using *in vivo* fluorescently labeled rFGF21 intravenously injected to GnRH::gfp mice, the authors show that peripheral FGF21 has a potential to reach the hypothalamic GnRH neuron terminals residing outside the blood–brain barrier (BBB), by extravasation through fenestrated vessels of the vascular organ of the lamina terminalis and the ME. The authors speculate that GnRH neuron terminals outside the BBB may perceive peripheral FGF21 to adapt GnRH secretion according to the metabolic state of the individuals.

They further demonstrate that FGF21/KLB/FGFR1 signaling plays an essential role in GnRH biology, which establishes a novel link between metabolism and reproduction in humans. Interestingly, a majority of patients with KLB mutations exhibit HH and, to some degree, metabolic defects (i.e., overweight, insulin resistance, and/or dyslipidemia) consistent with a metabolic role for this pathway. Persistence of defects after sex hormone replacement therapy has to be verified since this treatment usually improves metabolic parameters in CHH.

Previous studies had shown that FGF21 contributes to neuroendocrine control of female reproduction (Owen *et al*, 2013). Preventing reproduction during nutritional deprivation is an adaptive process that is essential for the survival of species. *Fgf21* transgenic mice exhibit GnRH deficiency with infertility by repressing the vasopressin‐kisspeptin pathway at the level of the suprachiasmatic nucleus in the hypothalamus (Chung *et al*, 2008). Both deficiency and excess of FGF21 may lead to defects in GnRH function. Such alterations of the FGF21/KLB/FGFR1 signaling pathway and especially mutations of KLB have to be searched in functional HH, like hypothalamic amenorrhea and obesity‐related HH. Indeed, a genetic basis for functional hypothalamic amenorrhea has been shown by the same group (Caronia *et al*, 2011).

The candidate gene approach used on CHH families is a winning strategy to identify new genes involved in IHH, allowing dissecting the FGFR1 molecular signaling pathway. It will gradually increase the proportion of patients for whom a genetic cause is identified. Interactome studies could be combined in the future to pinpoint potential candidate genes. To date, no pathogenic mutation is known for 50% of CHH patients, suggesting that additional mutations in currently unknown genes remain to be discovered.

## Conflict of interest

The author declares that she has no conflict of interest.
